# Latent profile analysis of compassion fatigue and its relationship with humanistic care ability among psychiatric nurses in Henan Province

**DOI:** 10.3389/fpubh.2026.1776812

**Published:** 2026-03-26

**Authors:** Junlei Zhang, Miao Yang, Fang Yan, Shouxun He, Chaona Shang, Yan Zhang

**Affiliations:** 1 Department of Child and Adolescent Psychiatry, The Second Affiliated Hospital of Henan Medical University, Xinxiang, China; 2 Key Laboratory of Child and Adolescent Psychiatry of Xinxiang City, Xinxiang, China; 3 School of Nursing, Henan Medical University, Xinxiang, China; 4 Department of Nursing, The Second Affiliated Hospital of Henan Medical University, Xinxiang, China

**Keywords:** Compassion fatigue, humanistic caring competence, Latent profile analysis, Nurse, psychiatric nursing

## Abstract

**Objective:**

To explore the potential categories of compassion fatigue in psychiatric nurses and their relationship with humanistic care ability.

**Methods:**

From December 2024 to January 2025, 1,093 nurses in the psychiatry departments of second-level and above psychiatric specialty hospitals and general hospitals in Henan Province were selected as study subjects by convenience sampling method, and they were investigated by using a general information questionnaire, the Chinese version of the Short Scale for compassion Fatigue, and the Chinese version of the Behavioral Scale for Caring. Mplus 8.3 (Muthén & Muthén, Los Angeles, CA, USA.) was used for potential profile analysis, and SPSS 27.0 software was used to compare the scores of psychiatric nurses with different categories of compassion fatigue on humanistic caring competence.

**Results:**

Psychiatric nurses' compassion fatigue could be categorized into 3 potential categories: low compassion fatigue-stable adaptation type (47.8%), Moderate compassion fatigue-fluctuating pressure type (42.3%), and High compassion fatigue-emotional exhaustion type (9.9%). The results of multiple logistic regression analysis showed that gender, health status, and whether or not they had received humanistic care training were influential factors affecting the potential profiles of compassion fatigue among psychiatric nurses. Comparison of the dimensions and total scores of the Caring Behavior Scale for psychiatric nurses with different compassion fatigue categories showed statistically significant differences (all *P* < 0.05). The higher the score of caring ability, the lower the score of compassion fatigue.

**Conclusion:**

This study identified three distinct latent profiles of compassion fatigue among psychiatric nurses using latent profile analysis: low compassion fatigue–stable adaptation type (47.8%, mean score 23.58 ± 9.72), Moderate compassion fatigue–fluctuating pressure type (42.3%, mean score 62.77 ± 10.21), and High compassion fatigue–emotional exhaustion type (9.9%, mean score 98.93 ± 15.34). Multivariate logistic regression revealed that male nurses were more likely to belong to the high compassion fatigue group (OR = 2.175, 95% CI: 1.261-3.750, *P* = 0.005), while good health status (OR = 0.052, 95% CI: 0.013-−0.209, *P* < 0.001) and prior humanistic care training (OR = 0.305, 95% CI: 0.180-−0.517, *P* < 0.001) significantly reduced the probability of being classified into the high compassion fatigue category. Furthermore, significant differences in humanistic caring ability were observed across the three profiles: nurses in the low compassion fatigue group scored highest on the Caring Behaviors Inventory (total score 90.43 ± 9.21), followed by the moderate group (82.64 ± 10.88) and the high group (80.64 ± 14.19) (*F* = 84.062, *P* < 0.001 for total score; all dimension scores also showed *P* < 0.001). These findings provide a granular understanding of the heterogeneity in compassion fatigue among psychiatric nurses and its strong association with humanistic caring competence. Nursing managers should develop targeted, profile-specific interventions-such as psychological resilience training for high-risk groups, stress management programs for the moderate group, and reinforcement of adaptive coping strategies for the low compassion fatigue group-to mitigate compassion fatigue and thereby enhance the quality of humanistic care.

## 1 Introduction

Compassion fatigue is a state of psychological and physiological exhaustion that occurs when healthcare professionals are exposed to the stress of traumatic events at work over a long period of time (1), which not only negatively affects the physical and mental health of healthcare professionals, but also negatively affects the quality of care and patient safety, and in severe cases, can lead to medical disputes (2). Psychiatric nurses face patients with mental disorders for a long time, and these patients often show abnormal behaviors such as emotional loss of control, attack and self-injury, resulting in nurses often being in a state of high alertness, and studies have shown (3, 4) that psychiatric nurses are more prone to compassion fatigue. Humanistic care competence refers to the ability to place human needs at the core, to take patients' rights and interests as the starting point, and to take into account the maintenance of patients' dignity and rights, quality and value of life in nursing practice, and this concept centrally embodies the essential characteristics of the nursing discipline (5). In the compassion fatigue assessment system, burnout and secondary traumatization constitute key assessment dimensions. Studies have shown (6, 7) that psychiatric nursing staff are exposed to high-risk work environments and high-intensity emotional investment over a long period of time, and the occurrence of burnout and secondary trauma can significantly weaken the group's ability to implement humanistic care, based on which, compassion fatigue may affect nurses' ability to implement humanistic care in their clinical work. At present, most of the existing studies on compassion fatigue among psychiatric nurses focus on the description of the current situation and the analysis of single influencing factors, but lack in-depth analysis from the perspective of group heterogeneity, and the relationship between the differences in group heterogeneity and the ability to implement humanistic care has not been studied. Guided by the Conservation of Resources theory (8), which holds that people are motivated to protect their existing resources, and that ongoing resource loss, especially chronic emotional exhaustion, can lead individuals to adopt adaptive strategies to preserve what remains-this study proposes a theoretical link between compassion fatigue and humanistic caring behavior. From this perspective, psychiatric nurses with higher levels of compassion fatigue may scale back their engagement in emotionally taxing caring behaviors as a way to avoid further resource depletion. Accordingly, different profiles of compassion fatigue may correspond to distinct patterns in how they perform humanistic care. Accordingly, this study tested the following hypotheses: ① Psychiatric nurses can be classified into distinct latent profiles based on compassion fatigue levels; ② These profiles differ significantly in terms of demographic and occupational factors; and ③ Nurses in higher fatigue profiles will demonstrate lower humanistic caring ability. The present study utilizes the potential profile analysis technique to systematically explore the heterogeneous characteristics of compassion fatigue in the psychiatric nurse group and its association with humanistic caring ability, this aims to provide a theoretical basis for the construction of targeted intervention strategies to reduce compassion fatigue and thus improve the level of humanistic caring practice in this group.

## 2 Objects and methods

### 2.1 Participants and procedures

In this study, from December 2024 to January 2025, psychiatric nursing staff of second-level and above psychiatric specialized medical institutions and general hospitals in Henan Province were selected as survey subjects through convenience sampling method. The specific process was as follows: conducted with the assistance of the Mental Health Nursing Professional Committee of the Henan Provincial Nursing Association, this committee contacted nursing directors from secondary-level and above mental health specialty hospitals and comprehensive hospitals within the province who were willing to participate, and these institutions were included in the study. Although this convenience sampling method ensured the feasibility of the study and an adequate sample size, it may introduce selection bias and limit the generalizability of the research findings to mental health nurses in other regions or healthcare settings. Furthermore, statistical control for potential clustering effects at the institutional level was not performed.

Sample inclusion criteria included: ① Having a valid nursing license and ≥1 year of psychiatric clinical nursing experience; ② Voluntarily signing an informed consent form to participate in the study. Sample exclusion criteria included: ① Nursing staff on maternity leave, sick leave, and other absences during the survey period; ② Nursing staff in the internship and advanced training stage.

This study is a cross-sectional study, including 16 items in the General Information Questionnaire for nursing staff, 13 items in the Short Scale of Compassion Fatigue, 24 items in the Scale of the Caring Behavior. The sample size was calculated using 5–10 times the maximum number of entries in the questionnaire. The Scale of the Caring Behavior had a total of 24 items, and considering 20% of invalid questionnaires, the sample size required for this survey was calculated to be 144-288 cases, and 1,125 psychiatric nurses who met the criteria were finally included.

### 2.2 Research tools

#### 2.2.1 General information questionnaire

According to the purpose, we designed a general information questionnaire through reviewing the literature, including gender, age, education level, marital status, health status, whether they are only child, number of children, monthly income, hospital level, title, position, nature of employment, years of work, nature of the department, shift, and whether they have received training in humanistic care.

#### 2.2.2 Chinese version of compassion fatigue short scale

The Compassion Fatigue Scale-revised version revised by Adams et al. (9) (2006) was used in this study. The scale has also been adapted and validated in different cultural contexts, ensuring its applicability across diverse populations (10, 11). It contains two dimensions of burnout (eight entries) and secondary trauma (five entries) on a 10-point Likert scale (1 = never, 10 = frequent), with a total score range of 13-130, with higher total scores indicating greater levels of compassion fatigue. In this study, Cronbach's α coefficient was 0.956.

#### 2.2.3 Chinese version of caring behaviors inventory

The Chinese version of Caring Behaviors Inventory was developed by Wolf (12) et al. in 1981, streamlined in 1994, revised by Wu Ying (13) in 2006, and Chineseized by Da Caojin et al. (14) in 2017 Revision. Contains three dimensions: support and assurance, knowledge and skills, and respect and connection. The entries of each dimension were distributed as nine items in the support and assurance dimension, fuve items in the knowledge and skills dimension, and 10 items in the respect and connection dimension, totaling 24 assessment indicators. The scale was based on a Likert 6-point scale (1 = never, 6 = always), with a theoretical total score range of 24-144, and its scale values were positively correlated with the level of humanistic care of nursing staff. The reliability test of the original scale showed that the overall Cronbach's α coefficient was 0.959. In this study, Cronbach's α coefficient was 0.981.

### 2.3 Data collection

Data were collected through online methods, and the informed consent form and scale were generated as QR codes relying on the Questionnaire Star platform. With the assistance of the Psychiatric Nursing Branch of Henan Provincial Nursing Society, the QR code was sent to the director of the nursing department of each hospital via WeChat with the consent and permission of the director of the nursing department of the surveyed hospitals, who then assigned a person responsible for filling out the questionnaire. To ensure the quality of the questionnaire and the accuracy of the data, the questionnaires were all set as mandatory questions, and the background settings were unified IP address could only be filled out once, excluding questionnaires with an answer time of less than 3 min or more than four-fifths of the same consecutive answer.

### 2.4 Ethical approval

This study was approved by the Institutional Review Board of The Second Affiliated Hospital of Xinxiang Medical University (Approval no. XYEFYLL-2024-102). All procedures strictly adhered to the ethical principles of the 1964 Helsinki Declaration and its subsequent amendments. Participants received a detailed information sheet outlining the study objectives, voluntary nature of participation, and data anonymity. Electronic informed consent was obtained prior to accessing the questionnaire.

### 2.5 Statistical analysis

This study was based on the Mplus 8.3 software platform to carry out a latent profile analysis to explore the latent category characteristics of compassion fatigue among psychiatric nurses through a progressive model fitting strategy. Starting from a single-category base model, the number of potential categories was incrementally increased, and the model fit was evaluated based on the following three types of indicators: (1) information evaluation indicators: including Akaike Information Criterion (AIC), Bayesian Information Criterion (BIC), and Corrected Bayesian Information Criterion (aBIC), whose decreasing values reflected the improvement of model fit; (2) discriminant effectiveness indicators: entropy as a parameter for classification accuracy, and its threshold value was 0.5. Discriminative efficiency index: entropy as a classification accuracy parameter, with a threshold range of 0-1, >0.8 characterizes the ideal classification accuracy; (3) Likelihood ratio test index: L-Mendell-Rubin test (LMR) and Bootstrap Likelihood Ratio Test (BLRT) are used, and if the test result reaches the significance threshold (*P* < 0.05), then it indicates that the k-category model has a better fitting effect compared with the k-1-category model. The data were analyzed based on SPSS 27. Measurement data in accordance with normal distribution were described by mean ± SD, and differences between groups were analyzed by one-way analysis of variance (ANOVA); categorical variables were described by frequency (percentage), and comparisons between groups were made by chi-square test. The influencing factors were analyzed using multinomial logistic regression, with the latent classes as the dependent variable (nominal variable) and the indicators showing statistically significant differences in the univariate analysis as the independent variables, to explore the determinants of different categories. The test level was set at α = 0.05.

## 3 Results

### 3.1 Participant general information

A total of 1,125 psychiatric nurses were surveyed in this study, 32 invalid questionnaires were excluded, 1,093 valid questionnaires were recovered, and the recovery rate of valid questionnaires was 97.16%. Of the 1,093 psychiatric nurses, 272 were male and 821 were female. The ages were concentrated in ≤ 29 years old and 30–39 years old. Educational level: 425 cases with specialties and below, 663 cases with bachelor's degree, and five cases with master's degree and above. Marital status: 686 cases were married, 407 cases were unmarried or divorced. Number of children: 0, 442 cases; 1–2, 631 cases; three, and above, 20 cases. Health status: 777 cases were good, 316 cases were average, poor and sick. Level of hospital: 205 cases of Grade 3A, 219 cases of Grade 3 comprehensive, 669 cases of Grade 2A and below. Years of working experience: ≤ 5 years 414 cases, 5~15 years 433 cases, ≥15 years 246 cases. Title: 377 cases of nurse, 364 cases of nurse practitioner, 331 cases of chief nurse, 21 cases of associate chief nurse and above. Position: nurse manager 74 cases, nursing team leader 47 cases, charge nurse 972 cases. Nature of department: closed 858 cases, open 235 cases. Shift: long day shift 406 cases, rotating night shift 687 cases. Received humanistic care training 805 cases, did not receive humanistic care training 288 cases.

### 3.2 Results and naming of potential profile analysis of compassion fatigue in psychiatric nurses

A total of 5 models were fitted, see Table 1. According to the model fitting indexes (AIC = 56,497.264, BIC = 56,767.085, aBIC = 56,595.569, Entropy = 0.965), the 3-category model was the best classification scheme. The Entropy value of 0.965 for model three is higher among the five models. This means that Model 3 is able to better distinguish different potential categories and the accuracy of classification is relatively more secure. In contrast, the Entropy values of Models 1, 2, 4, and 5 are lower than those of Model 3. The *P*-values of LMR and BLRT for Model 3 are less than 0.005, indicating that adding categories has an extremely significant improvement in the fitting of Model 3. The *P*-values of LMR and BLRT of model 5 are 0.3150 and 0.3187, respectively, which are greater than 0.05, indicating that the increase in categories does not significantly improve the fit of model 5; although the *P*-values of models 2 and 4 are less than 0.05, the improvement is not as great as that of model 3 in terms of significance; although from the point of view of AIC, BIC, and aBIC, the smaller the value of the model the better the model fit, and the value of model 5 has the smallest value in this regard. However, the model cannot be fully judged as superior or inferior based on these indicators alone. Model 3 is outstanding in terms of category differentiation (Entropy) and the significance of the increase in categories on the improvement of the fit (LMR, BLRT test), and by combining these two important factors, model 3 is significantly better than the other category models in terms of the overall performance, so model 3 is chosen.

**Table 1:** Indicators for fitting the model of potential categories of compassion fatigue in psychiatric nurses.

**Model**	**AIC**	**BIC**	**aBIC**	**Entropy**	***P***		**Class probabilities**
					**LMR**	**BLRT**	
1	67,023.803	67,153.717	67,071.135	—	—	—	—
2	59,003.316	59,203.183	59,076.134	0.962	< 0.001	< 0.001	0.544/0.456
3	56,497.264	56,767.085	56,595.569	0.965	0.0101	0.0104	0.478/0.423/0.099
4	55,225.062	55,564.837	55,348.853	0.939	0.0438	0.0455	0.403/0.283/0.252/0.062
5	54,228.492	54,638.219	54,377.769	0.949	0.3150	0.3187	0.248/0.361/0.058/0.262/0.071

**Figure 1:**
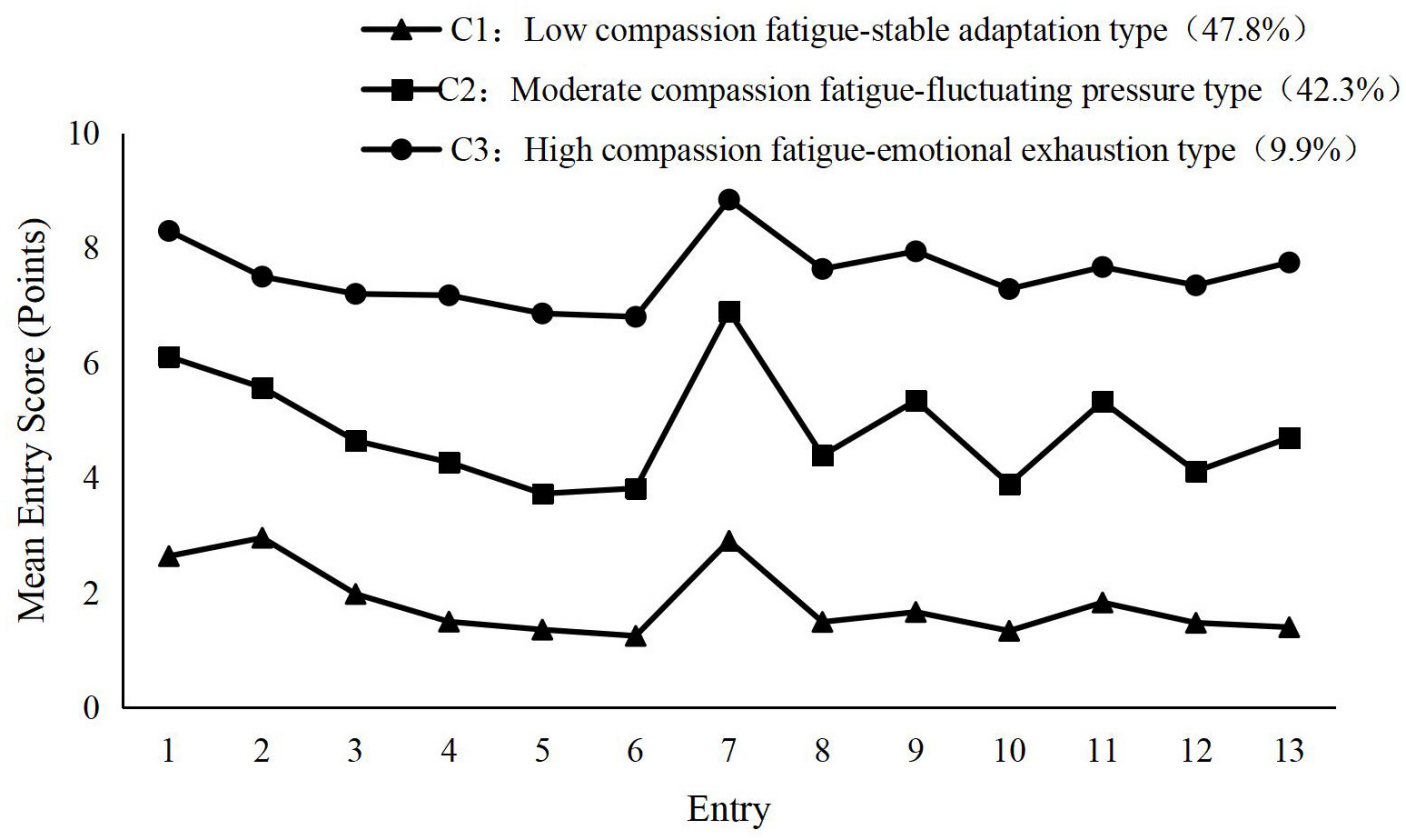
Distribution of entry scores for the three potential profiles of compassion fatigue in psychiatric nurses.

C1, C2, and C3 were named according to the distribution of each category of the model and the mean values of the entry scores, as shown in Figure 1. The mean values of the entry scores of C1 in the burnout and secondary trauma dimensions were all significantly lower than the critical values overall (M = 3.77). In the burnout dimension, the scores were generally at a low level, indicating that they were less likely to experience burnout-related symptoms; the same low scores in the secondary trauma dimension indicated that they were less affected by secondary trauma. So it is named Low compassion fatigue-stable adaptation type, with a score of (23.58 ± 9.72), accounting for 47.8% of the study participants.C2 There are obvious fluctuations in the scores of the burnout dimension and the secondary trauma dimension, with some of the entries scored elevated, especially in the burnout dimension, where there are some entries scored relatively prominently, showing a certain degree of burnout tendency; there were also score ups and downs in the secondary trauma dimension. The whole reflects a medium level of compassion fatigue, a certain amount of pressure at work, and fluctuating changes in the state, so it is named Moderate compassion fatigue—fluctuating pressure type, with a score of (62.77 ± 10.21), accounting for 42.3% of the research subjects. The mean value of the scores of the entries of C3 in the burnout and secondary trauma dimensions is generally high, and in the burnout dimension and secondary trauma dimension, most of the scores of the entries are at a high level, especially in the burnout dimension, which presents a persistent state of emotional depletion and is at a high level of compassion fatigue, so it is named as a High compassion fatigue-emotional exhaustion type, with a score of (98.93 ± 15.34) points and accounting for 9.9%.

### 3.3 One-way analysis of potential categories of compassion fatigue among psychiatric nurses

There was no statistically significant difference between the three potential categories of psychiatric nurses' compassion fatigue when comparing cultural level, marital status, whether they were only child, number of children, monthly income, hospital grade, title, position, nature of employment, years of experience, and nature of the department (all *P* > 0.05). The items with statistically significant differences are shown in Table 2.

**Table 2:** Univariate analysis of potential categories of compassion fatigue among psychiatric nurses.

**Item**	**Low compassion fatigue-stable adaptation type (*n* = 523)**	**Moderate compassion fatigue-fluctuating pressure type (*n* = 462)**	**High compassion fatigue-emotional exhaustion type (*n* = 108)**	**χ^2^**	***P***
**Gender**					0.016
Male	116 (22.18)	118 (25.54)	38 (35.19)	8.284	
15.6-7.4,-1.3498ptFemale	407 (77.82)	344 (74.46)	70 (64.81)		
**Health status**					< 0.001
Good	439 (83.94)	290 (62.77)	48 (44.44)	103.353	
Fair	81 (15.49)	149 (32.25)	49 (45.37)		
15.6-7.4,-1.3498ptPoor/ill	3 (0.57)	23 (4.98)	11 (10.19)		
**Shift**					0.022
Long day shift	215 (41.11)	159 (34.42)	32 (29.63)	7.607	
28-7.4,-1.3498ptRotating night shift	308 (58.89)	303 (65.58)	76 (70.37)		
**Whether trained**					< 0.001
**in humanistic care**					
Yes	439 (83.94)	313 (67.75)	53 (49.07)	70.431	
No	84 (16.06)	149 (32.25)	55 (50.93)		

### 3.4 Multifactorial analysis of potential categories of compassion fatigue among psychiatric nurses

Logistic regression analysis was conducted with psychiatric nurses' compassion fatigue category as the dependent variable and the indicators with statistically significant differences in the univariate analysis as the independent variables. C1 (Low compassion fatigue-stable adaptation type), C2 (Moderate compassion fatigue-fluctuating pressure type), and C3 (High compassion fatigue-emotional exhaustion type) were assigned as 1, 2, and 3, respectively. The independent variables were assigned as follows: gender: male = 1, female = 2; health status: good=1, fair = 2, poor/diseased = 3; shifts, long day shifts = 1, rotating night shifts = 2; and whether or not they had received humanistic care training: yes = 1, no = 2. It should be noted that latent classes are nominal variables, and the class codes (C1, C2, C3) serve solely as analytical identifiers, with no implication of ordinal or hierarchical relationships between the categories. Logistic regression analysis showed that gender, health status, and whether or not they had received humanistic training were influential factors in the potential profile of compassion fatigue among psychiatric nurses (*P* < 0.05). The results are shown in Table 3.

**Table 3:** Logistic regression analysis results of the three potential profiles of psychiatric nurses' compassion fatigue influencing factors.

**Group comparison**	**Item**	**β**	***SE***	**Wald χ^2^**	***P***	***OR***	**95% *CI***
C1 vs. C2^*^	Sex						
	Male	0.347	0.1604	4.672	0.031	1.415	1.033~1.937
	Health status						
	Good	−2.265	0.6243	13.167	< 0.001	0.104	0.031~0.353
	General	−1.365	0.6345	4.628	0.031	0.255	0.074~0.886
	Whether they have received training in humanistic care						
	Yes	−0.711	0.1680	17.896	< 0.001	0.491	0.354~0.683
C1 vs. C3^*^	Sex						
	Male	0.777	0.2780	7.811	0.005	2.175	1.261~3.750
	health status						
	Good	−2.956	0.7091	17.383	< 0.001	0.052	0.013~0.209
	General	−1.438	0.7186	4.005	0.045	0.237	0.058~0.971
	Whether they have received training in humanistic care						
	Yes	−1.189	0.2695	19.461	< 0.001	0.305	0.180~0.517
C2 vs. C3^*^	Health status						
	Good	−0.863	0.4252	4.120	0.042	0.422	0.183~0.971
	Whether they have received training in humanistic care						
	Yes	−0.641	0.2323	7.611	0.006	0.527	0.334~0.831

C1 is the low compassion fatigue-stable adaptation group, C2 is the compassion fatigue-fluctuating stress group, and C3 is the high compassion fatigue-emotional exhaustion group. The labeled ^*^ group is the reference group.

### 3.5 Comparison of differences in caring behaviors of psychiatric nurses with different potential categories of compassion fatigue

Comparison of the total scores of caring behaviors and the scores of each dimension of the three different compassion fatigue potential categories extracted by the potential profile analysis method, the differences were statistically significant (*P* < 0.001), see Table 4.

**Table 4:** Comparison of differences in caring behavior scores of psychiatric nurses with different potential categories of compassion fatigue.

**Group**	**Support and reassurance**	**Knowledge & skills**	**Respect and connection**	**Total score**
Low compassion fatigue-stable adaptation type	3.81 ± 0.39	3.80 ± 0.38	3.71 ± 0.44	90.43 ± 9.21
Moderate compassion fatigue-fluctuating pressure type	3.52 ± 0.47	3.49 ± 0.48	3.35 ± 0.50	82.64 ± 10.88
High compassion fatigue-emotional exhaustion type	3.42 ± 0.61	3.40 ± 0.62	3.29 ± 0.62	80.64 ± 14.19
*F*	66.748	73.787	83.397	84.062
*P*	< 0.001	*P* < 0.001	*P* < 0.001	*P* < 0.001

## 4 Discussion

### 4.1 Characterization of potential categories of compassion fatigue among psychiatric nurses

Using potential profile analysis in this study, it was found that psychiatric nurses' compassion fatigue could be divided into three potential categories: low compassion fatigue-stable adaptation type, Moderate compassion fatigue-fluctuating pressure type, and High compassion fatigue-emotional exhaustion type, and there were significant individual differences.

Low compassion fatigue-stable adaptation type psychiatric nurses accounted for 47.8% of the total sample, nearly half of all study participants. This suggests that nurses in this group are better able to cope with work-related stress and challenges. They can effectively manage the traumatic emotions and psychiatric symptoms encountered in their work, maintaining high psychological resilience. This may be associated with the widespread implementation of the magnet hospital management model in nursing practice in recent years, which has created a supportive working environment, enhanced nurses' professional identity, and boosted their work motivation (15, 16). These nurses are an important stabilizing force and source of positive energy within the department. Their presence helps maintain the morale of the nursing team and the continuity of service quality. For this subgroup of psychiatric nurses, it is recommended that healthcare institutions expand their access to participate in departmental management decisions as a way to strengthen the sense of professional belonging and maintain enthusiasm for professional services.

The proportion of psychiatric nurses with Moderate compassion fatigue-fluctuating pressure type was 42.3%, which is consistent with the study of Ge et al. (17). In this subgroup, the scores for each dimension of the compassion fatigue scale were intermediate between those of the C1 and C3 groups, with moderate levels of burnout and secondary traumatization, indicating that moderate compassion fatigue is prevalent among psychiatric nurses. Nurses of this type are in a state of dynamic balance. Their level of compassion fatigue fluctuates with workloads, patient behavior incidents, shift cycles, or personal conditions. During periods of relatively low stress, they can demonstrate caregiving abilities comparable to those of low-fatigue nurses; however, after experiencing high-pressure situations such as heavy work intensity, patient aggression, or self-harm events, their emotional resource depletion intensifies, potentially leading to temporary emotional detachment, reduced caregiving behaviors, or decreased work engagement. The professional status of these nurses is somewhat malleable, making them a key target group for intervention. Appropriate support and management strategies can help them transition toward a stable adaptive type. It is suggested that management can design targeted intervention strategies based on the individual characteristics and professional strengths of nurses: ① Build a career development support system to provide personalized career navigation and competency enhancement programs; ② Establish a mechanism for psychological resilience. It is recommended to organize and maintain training programs to strengthen the psychological resilience of health workers who are trying to provide compassionate care under intense and stressful working conditions (18). Professional support such as regular psychological supervision and post-traumatic growth workshops can systematically alleviate empathic stress; ③ Implement a dynamic incentive mechanism, match diverse development paths based on job competency, and activate the intrinsic driving force of career development.

The percentage of psychiatric nurses with High compassion fatigue-emotional exhaustion type was 9.9%, which is similar to the study of Yin et al. (19). Psychiatric nurses in this subgroup accumulated unprocessed emotional stress due to excessive compassion, which eventually erupted in the form of somatic exhaustion. In clinical practice, they often exhibit persistent emotional exhaustion, growing indifference toward work, and diminished or avoidant responses to patients' suffering, sometimes accompanied by physical and psychological symptoms (such as insomnia, irritability, or somatic discomfort). Their willingness and capacity to provide humanistic care are significantly impaired, leading them to adopt more task-oriented, procedural nursing approaches and weakening emotional connections with patients. This not only affects the quality of care and patient safety but also substantially increases their own risk of leaving the profession. This group represents a key population requiring priority identification and intensive psychological occupational intervention. Nursing managers should routinely review the work intensity of psychiatric nurses in this subgroup to avoid excessive work pressure; develop both personalized intervention programs, such as mindfulness interventions and emotion focused training, these methods have been proven in previous studies to effectively reduce compassion fatigue among healthcare professionals (20–23); and organize regular group activities in the department, such as outings for picnics, K-songs, and psychological games, to help the nurses release their stress and alleviate their compassion fatigue.

### 4.2 Analysis of factors influencing the potential categories of compassion fatigue among psychiatric nurses

#### 4.2.1 Male nurses' compassion fatigue is more likely to be categorized into the high compassion fatigue-emotional exhaustion type

The results of multivariate logistic regression showed that male nurses were more likely to be categorized into the High compassion fatigue-emotional exhaustion type, which is consistent with the study of Roy et al. (24). The reasons were analyzed as, firstly, regarding social expectations, traditional gender roles may lead men to be more inclined to inhibit emotional expression and reduce emotional involvement with patients, secondly, in terms of coping styles, male nurses may tend to use problem-oriented coping styles rather than emotional immersion, thus avoiding emotional depletion, and finally, with respect to psychological resilience, research has shown (25) that psychological resilience is a protective compassion fatigue factor, and male nurses may reduce compassion fatigue due to greater psychological resilience.

#### 4.2.2 Psychiatric nurses in good health are less likely to be categorized as High compassion fatigue-emotional exhaustion type

Multiple logistic regression analysis showed that psychiatric nurses with good self-reported health were more likely to be categorized into the Moderate compassion fatigue-fluctuating pressure type, which is consistent with the study of Cho et al. (26). Psychiatric nurses in good health are not only able to fully utilize protective resources for self-care, but also understand the patient's experience of illness with abundant energy and are able to provide high-quality care to their patients, thus allowing themselves to avoid exposure to traumatic scenarios. Managers are prompted to pay attention to nurses in ill health and arrange paid leave when necessary to relieve their stress and provide them with effective support in clinical practice.

#### 4.2.3 Humanistic care training significantly reduces compassion fatigue

The results of this study show that the probability of psychiatric nurses who have received humanistic care training entering the High compassion fatigue-emotional exhaustion type group is significantly reduced. This finding directly supports the implementation of humanistic care training as a protective strategy. Humanistic care training can help nurses master compassion skills and improve their compassion ability through systematic theoretical learning and scenario simulation (27, 28). Compassion, as an important component of nurse-patient communication, can not only bring positive impacts to patients, and enhance their treatment adherence, but also promote the professional growth of nurses, and improve their job satisfaction and sense of professional accomplishment. Previous studies have demonstrated that the higher the compassion ability of clinical nurses, the lower the degree of compassion fatigue (29). Therefore, it is recommended that managers optimize the content of humanistic care training by incorporating common psychiatric scenarios (e.g., dealing with patient aggression, self-injury, and suicide) and self-care skills (30) (e.g., positive stress reduction). Enhancing nurses' compassion ability and skills reduces emotional transition consumption, thus alleviating compassion fatigue.

### 4.3 Differences in humanistic caring ability scores of psychiatric nurses with different compassion fatigue categories

Research (31, 32) shows that compassion fatigue is significantly negatively correlated with humanistic care. In this study, Low compassion fatigue-stable adaptation type nurses had the highest humanistic caring ability scores, Moderate compassion fatigue-fluctuating pressure type nurses had the second highest scores, and High compassion fatigue-emotional exhaustion type nurses had the lowest scores, and there was a significant difference between the three types of psychiatric nurses in terms of total humanistic caring ability and scores of dimensions (*P* < 0.05), indicating that decreasing the level of compassion fatigue can directly or indirectly affect psychiatric nurses' humanistic caring ability. According to the conservation of resources theory within the compassion fatigue model (8) long-term exposure to patient aggression, self-harm, or suicidal behaviors requires psychiatric nurses to maintain continuous high-intensity emotional input. Such prolonged emotional depletion tends to induce compassion fatigue. To avoid further overdraft of psychological and social resources, nurses may adopt strategies that reduce caring behaviors toward patients. In addition, the psychiatric occupational environment is unique, and psychiatric nurses have to be in a state of high alert at work under the multiple pressures of long-term violence and physical threats, which reduces work motivation, compassion fatigue, and then reduces humanistic caring behaviors. Low compassion fatigue psychiatric nurses have a high sense of professional identity (33), which allows them to synthesize their cognition, emotions and behaviors at work and apply them to nursing care, and a positive sense of professional identity enables nurses to form excellent professional qualities, which in turn facilitates humanistic caring competence (34). Therefore, it is recommended that nursing managers carry out regular psychological supervision, peer support, reasonable leave, training in communication skills (e.g., nonviolent communication), optimization of workflow, and other management interventions for psychiatric nurses, so as to effectively control the level of occupational emotional exhaustion of psychiatric nursing staff. At the same time, we should pay attention to the demonstration effect of the “low compassion fatigue-stable adaptation” nursing staff, and through the establishment of a standardized self-adaptation training program, we can promote the benign transfer of emotional management skills among this group, reduce the compassion fatigue level of all nursing staff, and then enhance the ability of humanistic care.

## 5 Conclusion

In this study, compassion fatigue among psychiatric nurses was categorized into three potential categories: low compassion fatigue-stable adaptation type (47.8%), Moderate compassion fatigue-fluctuating pressure type (42.3%), and High compassion fatigue-emotional exhaustion type (9.9%) through potential profile analysis. Gender, health status, and whether or not they have received humanistic training are the influencing factors of potential profiles of compassion fatigue among psychiatric nurses. Specifically, male nurses are more likely to be categorized into the high fatigue group; those with good health status are more likely to belong to the moderate fatigue category rather than the high fatigue category; receiving humanistic care training significantly reduces the risk of being classified into the high fatigue category. Meanwhile, this study analyzed the differences in humanistic caring ability of psychiatric nurses with different categories of compassion fatigue, there were significant differences in the total score of humanistic care ability and the scores of each dimension among nurses with different fatigue categories (*P* < 0.001). The low fatigue group had the highest care ability (90.43 ± 9.21), followed by the moderate fatigue group (82.64 ± 10.88), and the high fatigue group had the lowest (80.64 ± 14.19). This indicates that controlling the level of compassion fatigue can help improve the humanistic care practice ability of psychiatric nurses. Which provided a theoretical basis for the relationship between the level of compassion fatigue and humanistic caring ability of psychiatric nurses.

## 6 Limitations

This study has several limitations that should be acknowledged. First, the geographical scope was restricted to Henan Province, which may limit the generalizability of the findings. Second, as a cross-sectional investigation, it could only reveal associations between variables rather than establish a causal relationship between compassion fatigue and humanistic care ability among psychiatric nurses. Third, the reliance on self-reported measures may introduce social desirability and recall bias, particularly for humanistic care behaviors. Fourth, organizational-level factors such as staffing ratios, workload, and leadership style were not assessed, which could influence compassion fatigue profiles. Finally, the temporal stability of the identified latent classes was not examined. Additionally, this design fails to capture the dynamic changes in compassion fatigue over time. Future research should address these limitations by conducting multi-center, longitudinal studies with larger sample sizes to further validate the conclusions drawn from the present study.

## Data Availability

The original contributions presented in the study are included in the article/supplementary material, further inquiries can be directed to the corresponding author.
